# Prognoses for diagnoses: medical search online and “cyberchondria”

**DOI:** 10.1186/1753-6561-6-S4-P30

**Published:** 2012-07-09

**Authors:** M Aiken, G Kirwan

**Affiliations:** 1 Royal College of Surgeons in Ireland, Ireland

## Introduction

The Internet is a source of valuable medical information, however the Web has the potential to increase anxieties of people with no medical training when employed as a diagnostic procedure (White & Horvitz, 2009). Anxiety induced as a result of health related search online is an increasingly differentiated activity ( Fox et al., 2000; Feldman, 2000; Lewis, 2006; Belling, 2006; Ravdin, 2008; White & Horvitz, 2009) and known in the field of cyberpsychology as cyberchondria. This literature review aims to review research studies that have investigated medical information seeking online in the general population.

## Methods

Research journals from 2000-2011 were selected and studied to identify consistent and contrasting views. Themes identified were as follows; impact of technology on health related information seeking; the role of symptom search concerning self-diagnostics online; knowledge empowered challenges to medical opinion; threats to traditional the doctor-patient relationships; consideration of the 'worried well', health anxiety, hypochondria, cyberchondria and online escalation; DSM-V task force recommendations regarding revisions to somatoform disorder classification; exploration of virtual factitious disorders; anonymity, self-disclosure, disinhibition, altruism and reassurance/support considered in the context of medical chat room forums online, these themes will be discussed in this literature review.

## Results

Literature review indicates that health-related search technology impacts how information is disseminated, and can cause unnecessary anxiety (White & Horvitz, 2009). Knowledge, empowerment (Bastian, 2003), support, reassurance (Sillence & Briggs, 2007) and altruism (Adar & Huberman, 2000) may be positive aspects regarding medical search online, however the literature (Belling, 2006; Lewis, 2006; Ravdin, 2008; White & Horvitz, 2009 ) indicates that anxiety is likely to be a consequence of same, additionally emergence of the 'Google stack' in the consultation process is impacting on traditional doctor patient relationships ( Belling, 2006; Lewis, 2006).

## Conclusions

These findings are relevant for healthcare professionals, particularly regarding patient care and management, the Internet's capacity as an omnipresent delivery mechanism for medical search, coupled with known propensity to escalate online, makes a strong case for further study of this subject.

